# Proteomic Analysis of Ovarian Cancer Proximal Fluids: Validation of Elevated Peroxiredoxin 1 in Patient Peripheral Circulation

**DOI:** 10.1371/journal.pone.0025056

**Published:** 2011-09-30

**Authors:** Ebony R. Hoskins, Brian L. Hood, Mai Sun, Thomas C. Krivak, Robert P. Edwards, Thomas P. Conrads

**Affiliations:** 1 Division of Gynecologic Oncology, Department of Obstetrics, Gynecology, and Reproductive Sciences at Magee-Womens Hospital of the University of Pittsburgh School of Medicine, University of Pittsburgh School of Medicine, Pittsburgh, Pennsylvania, United States of America; 2 Gynecologic Cancer Center of Excellence, Women's Health Integrated Research Center at Inova Health System, Annandale, Virginia, United States of America; 3 University of Pittsburgh Cancer Institute, Pittsburgh, Pennsylvania, United States of America; University of Nebraska Medical Center, United States of America

## Abstract

**Background:**

Epithelial ovarian cancer (EOC) is the deadliest gynecologic malignancy in the United States. Unfortunately, a validated protein biomarker-screening test to detect early stage disease from peripheral blood has not yet been developed. The present investigation assesses the ability to identify tumor relevant proteins from ovarian cancer proximal fluids, including tissue interstitial fluid (TIF) and corresponding ascites, from patients with papillary serous EOC and translates these findings to targeted blood-based immunoassays.

**Methodology/Principal Findings:**

Paired TIF and ascites collected from four papillary serous EOC patients at the time of surgery underwent immunodepletion, resolution by 1D gel electrophoresis and in-gel digestion for analysis by liquid chromatography-tandem mass spectrometry, which resulted in an aggregate identification of 569 and 171 proteins from TIF and ascites, respectively. Of these, peroxiredoxin I (PRDX1) was selected for validation in serum by ELISA and demonstrated to be present and significantly elevated (p = 0.0188) in 20 EOC patients with a mean level of 26.0 ng/mL (±9.27 SEM) as compared to 4.19 ng/mL (±2.58 SEM) from 16 patients with normal/benign ovarian pathology.

**Conclusions/Significance:**

We have utilized a workflow for harvesting EOC-relevant proximal biofluids, including TIF and ascites, for proteomic analysis. Among the differentially abundant proteins identified from these proximal fluids, PRDX1 was demonstrated to be present in serum and shown by ELISA to be elevated by nearly 6-fold in papillary serous EOC patients relative to normal/benign patients. Our findings demonstrate the facile ability to discover potential EOC-relevant proteins in proximal fluids and confirm their presence in peripheral blood serum. In addition, our finding of elevated levels of PRDX1 in the serum of EOC patients versus normal/benign patients warrants further evaluation as a tumor specific biomarker for EOC.

## Introduction

Approximately 22,220 women are diagnosed annually with epithelial ovarian cancer (EOC) and over 13,000 succumb to this disease [1]. Women with EOC have a 5-year survival rate of 18–34%, where the poor prognosis is primarily due to the fact that the majority of patients present with advanced stage disease [2]. Detection of early stage disease with an optimal screening test would have a positive impact on overall survival; however, there are no available biomarkers that are sufficiently sensitive or specific for deployment of a suitable test to be of utility for screening the general population. Mucin 16, also known as cancer antigen (CA)-125, was discovered by antibody production of tumor xenografts [3] and is readily detected in serum. An FDA-approved CA-125 immunoassay is currently in use to monitor disease response to chemotherapeutic treatment and recurrence; however, measurement of CA-125 is ineffective for screening (an off-label use) for EOC in the general population [4]. While immunodetection of tumor-associated antigens has contributed to the knowledge of the natural history of the disease, their measures have yet to consistently identify early stage EOC.

High throughput gene expression analysis of normal ovarian tissue compared to EOC has revealed unique gene signatures expressed in EOC. Although these signatures have provided further insight into the molecular biology of EOC, gene expression does not consistently correlate with protein abundance [5], particularly in peripheral blood, which represents the most clinically relevant sample source for routine assay development and measurement [6]. While proteomic profiling of serum represents an attractive approach for biomarker discovery, this strategy is analytically challenging due to the high dynamic range of protein concentration in this sample, which impedes discovery of lower abundance, tumor-related proteins. Arising from the inherent analytical challenges related to serum proteomics, proximal fluids have gained increasing attention for conducting candidate protein biomarker discovery [7]. In the case of EOC, ascites represents an attractive sample source for candidate discovery as it bathes non-adherent cancer cells and adjacent mesothelial cells and contains abundant information including growth, survival and metastasis signaling factors [8]. While ascites is an important medium for conducting protein biomarker discovery investigations, much like serum, it also contains proteins present across a high dynamic range of concentration requiring various fractionation techniques to in order to identify lower abundant proteins [9].

Tissue interstitial fluid (TIF) is another sample that has gained increasing attention as a substrate from which candidate protein biomarker discovery from tumor tissue can be conducted. Tissue interstitial fluid is a protein rich sample source that is hypothesized to contain secreted, shed and/or effluxed proteins from the tumor and neighboring stroma. Celis et al. were the first to investigate proteins from breast cancer and normal TIF [10], where it was demonstrated that proteins identified by MS-based proteomics could be externally validated in a tissue microarray containing over 70 breast carcinoma tissue samples [11]. In a more recent investigation, an MS-based proteomics workflow for identifying differentially abundant proteins in renal cell carcinoma (RCC) demonstrated elevated levels of enolase 2 (ENO2) and thrombospondin-1 (TSP1) in tumor TIF. These were also verified as present and at elevated abundance in RCC patient serum samples as compared to control serum [12]. This investigation demonstrated that proteins discovered in TIF possess a high propensity for translation to serum-based assays.

In the current study, we utilized an MS-based proteomics workflow to identify tumor specific proteins from TIF and ascites from EOC patients. Among the differentially abundant proteins identified from these proximal fluids, peroxiredoxin 1 (PRDX1) was demonstrated to be present in serum and shown by ELISA to be elevated by nearly 6-fold in papillary serous EOC patients relative to normal/benign patients. Our study adds further support to the hypothesis that TIF represents an attractive proximal biofluid for conducting candidate biomarker discovery for the eventual early detection of EOC.

## Results

**Figure 1:**
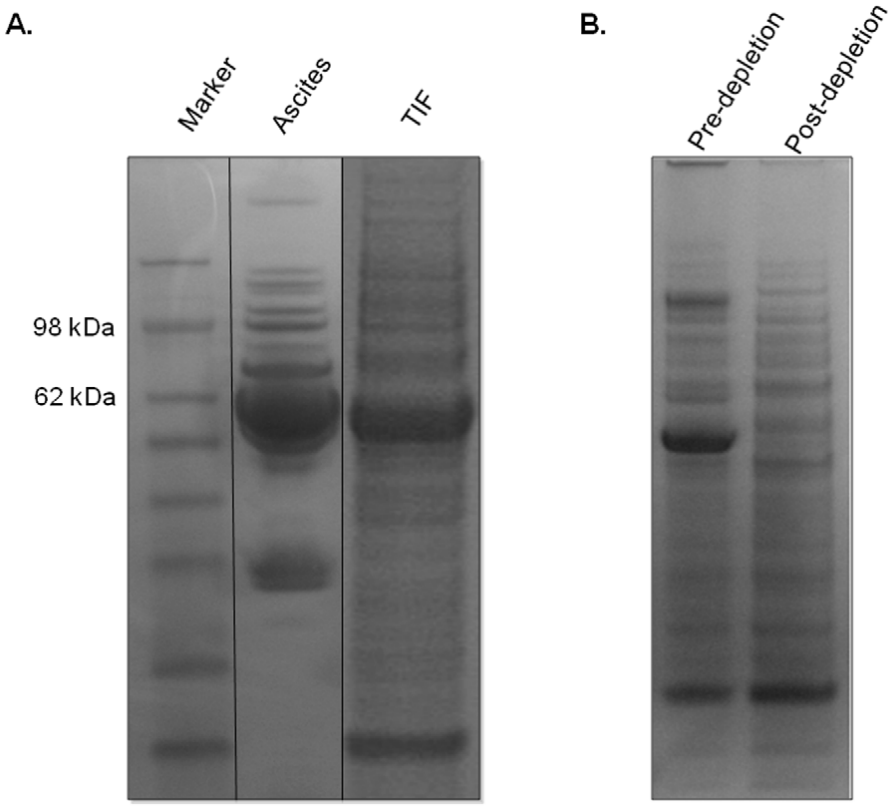
1D-PAGE of TIF and Ascites. (A) Representative ascites and TIF samples illustrating the presence of abundant protein species in both samples as well as the complexity of the harvested TIF sample. (B) A representative TIF sample before and after immunodepletion with the Agilent MARS Hu-14 immunoaffinity column.

The protein content recovered from TIF and ascites was visualized by resolution on a 1D-PAGE gel (Figure 1). A significant amount of human serum albumin (HSA) was present in both samples, although at lower levels in TIF than ascites. This greater level of HSA in ascites is significant as it effectively increases the overall dynamic range of protein concentration between the highest (e.g. HSA) and lowest abundant proteins in this sample type. A greater representation of the proteome is apparent in the TIF sample due to the lower overall contribution of HSA to the total protein content. Given the different contribution of HSA in these two samples, our investigation utilized an affinity-based immunodepletion protocol to remove the classical highly abundant serum proteins present in each sample. This methodology resulted in an efficient removal of a number of highly abundant proteins as shown for a TIF sample (Figure 1), allowing for a more complete comparison of the proteomic content of ascites and TIF following LC-MS/MS analysis.

**Figure 2:**
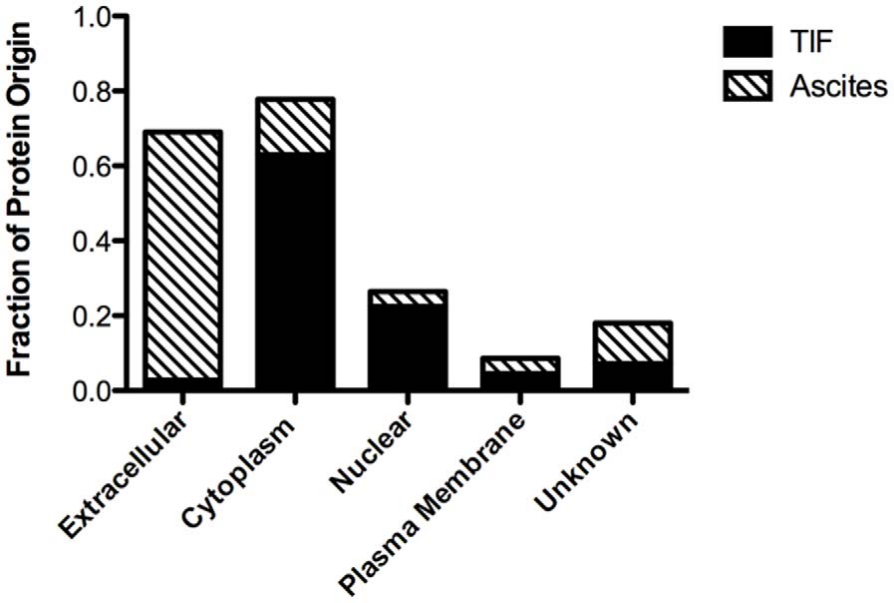
Gene ontology analysis for the origin of protein localization. Tissue interstitial fluid and ascites proteins which were identified by two or more spectral counts in each biofluid, respectfully, were analyzed for cellular compartmentalization with Ingenuity Pathway Analysis (IPA). Nearly 85% of TIF proteins originated in cytoplasm and/or nucleus. Ascites had a serum-like protein profile with 66% of proteins being extracellular in origin.

Paired ascites and TIF were harvested from four patients (Table 1), immunodepleted and equivalent protein amounts of each resolved by 1D-PAGE. Each gel lane was cut into five equivalent slices, in-gel digested and the peptide digests analyzed by LC-MS/MS in duplicate, resulting in 10,274 proteins identified across all samples, including those identified by a single peptide. We chose to impose an exclusion criterion such that only those proteins represented by two spectral counts or more across all four patient samples from either TIF or ascites were retained for the comparative proteomic data analysis, which resulted in a data set comprised of 569 and 171 proteins identified in all four TIF and ascites patient samples, respectively (Table S1; the raw LC-MS/MS data files are available for download from the Tranche Data Repository at https://proteomecommons.org/tranche/). Ingenuity Pathway Analysis annotation of the cellular localization of proteins identified revealed a large enrichment in cytoplasmic- and nuclear-derived proteins in TIF relative to ascites (Figure 2). Not surprisingly, a high proportion of extracellular proteins were present in ascites as compared with TIF. Extracellular proteins dominated the common proteins identified, with the vast majority derived from ascites. Subcellular proteins generally originated from TIF; these proteins were in higher concentration in TIF with diminished levels in ascites. This contrasting difference in the origin of cellular localization suggests that TIF contains proteins highly reflective of the cellular composition of the tissue microenvironment.

**Table 1:** Clinicopathological features of ovarian cancer patients in the discovery set.

Patient	Age	Race	Histology	Stage	Neoadjuvant Chemotherapy
1	49	Caucasian	Papillary Serous	IV	None
2	39	Caucasian	Papillary Serous	IV	None
3	67	Caucasian	Papillary Serous	IIC	None
4	61	Caucasian	Papillary Serous	IIIC	None

**Figure 3:**
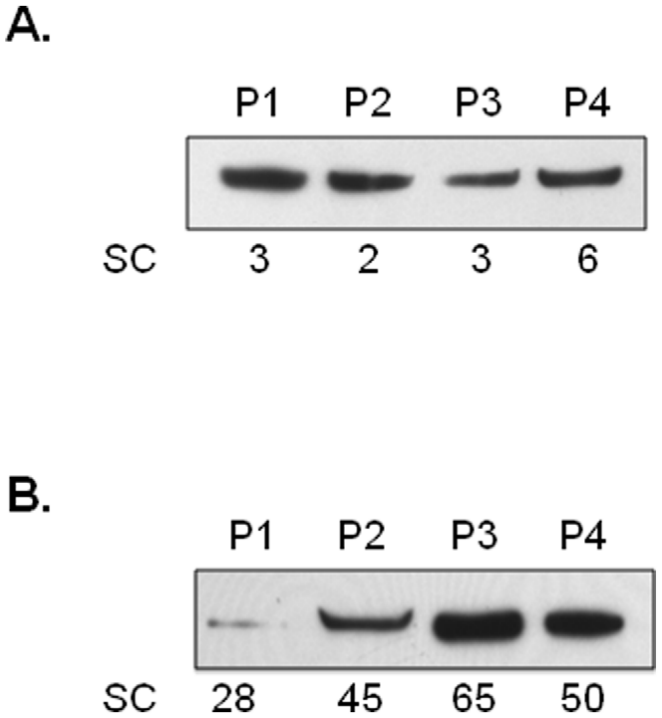
Cytoplasmic-derived peroxiredoxin 1 (PRDX1) is confirmed in TIF and Ascites. Peroxiredoxin 1 verification via western blot analysis in ascites (A) and TIF (B) recapitulates the results obtained by the spectral count (SC) values measured from the liquid chromatography-tandem mass spectrometry analysis of these samples.

**Figure 4:**
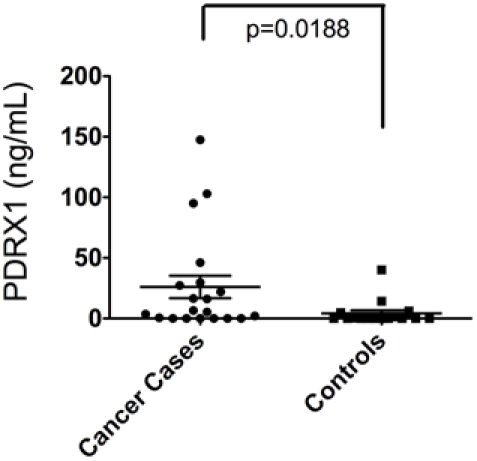
Peroxiredoxin 1 is elevated in serum from ovarian cancer patients. Serum from 20 patients diagnosed with Stage IIC or higher ovarian cancer (cases) and serum from 20 patients with benign ovarian pathology (controls) was evaluated for PDRX1 presence and quantity by ELISA. There was a statistically significant difference between the mean PDRX1 levels (p = 0.0188) in the two groups.

Among those proteins identified, peroxiredoxin 1 (PRDX1) was identified in greater abundance (an average of 13-fold greater spectral count) in TIF samples as compared to ascites. We confirmed the presence and differential abundance of PRDX1 in both biospecimens by western blot (Figure 3). While PDRX1 is routinely identified and studied in tissue, we sought to determine if the abundance of PRDX1 was elevated in serum from a naïve cohort (e.g. not part of our original discovery set) of 20 patients with stage II or higher EOC (Table 2) as compared to patients with a benign ovarian pathology (Table 3). The mean level of PRDX1 from these 20 EOC patients was determined to be 26.0 ng/mL (±9.27 SEM) and 4.19 ng/mL (±2.58 SEM) from the 16 control/benign ovarian populations as measured using a commercially available PDRX1 ELISA. This approximate 6-fold greater abundance of PDRX1 in serum of EOC patients was found to be statistically significant (Figure 4, p = 0.0188).

**Table 2:** Clinicopathological features of epithelial ovarian cancer patients in the validation set.

Patient	Age	Race	Histology	Stage	Medical Comorbidities
1	74	Caucasian	Papillary Serous	IIIC	
2	85	Caucasian	Papillary Serous	IIIC	
3	69	Caucasian	Papillary Serous	IIIC	Neoadjuvant chemotherapy
4	70	Caucasian	Papillary Serous	IIIC	
5	86	Caucasian	Papillary Serous	IIIC	
6	48	Caucasian	Papillary Serous	IIIC	
7	66	Caucasian	Papillary Serous	IIIC	
8	64	Caucasian	Papillary Serous	IIIC	
9	80	Caucasian	Papillary Serous	IIIC	
10	70	Caucasian	Papillary Serous	IIIC	
11	57	Caucasian	Papillary Serous	IIIC	
12	68	Caucasian	Papillary Serous	IIIC	Suboptimally debulked
13	48	Caucasian	Papillary Serous	IV	
14	71	Caucasian	Papillary Serous	IIIC	
15	77	Caucasian	Papillary Serous	IIIC	Focal clear cell differentiation
16	54	Caucasian	Papillary Serous	IIIC	
17	60	Caucasian	Papillary Serous	IIIC	
18	75	Caucasian	Papillary Serous	IIIC	
19	84	Caucasian	Papillary Serous	IIIC	Suboptimally debulked
20	48	Caucasian	Papillary Serous	IIIC	

**Table 3:** Clinicopathological features of patients with benign ovarian pathology in the validation set.

Patient	Age	Race	Ovarian Pathology	Medical Comorbidities
1	42	Caucasian	Normal	
2	38	Caucasian	Normal	T1c N0 MO Infiltrating Ductal Carcinoma
3	48	Caucasian	Normal	Family History of Breast and Ovarian Cancer
4	43	Caucasian	Mucinous Cystadenofibroma	
5	37	Caucasian	Endometriosis	Family History of Ovarian Cancer
6	72	Caucasian	Normal	Stage IA Grade3 Endometrial Cancer
7	69	Caucasian	Cystadenofibroma	
8	47	Caucasian	Normal	Complex Atypical Hyperplasia
9	37	Caucasian	R Mucinous Adenoma/L Endometrioma	
10	35	Caucasian	Normal	T2 N0 MO Infiltrating Ductal Carcinoma/Brca 1 Mutation
11	77	Caucasian	Normal	
12	58	Caucasian	Fibrothecoma	
13	70	Caucasian	Bilateral Serous Cystadenofibroma	
14	42	Caucasian	Normal	
15	52	Caucasian	Fibrothecoma	
16	59	Caucasian	Fibroma	

## Discussion

Presentation with abdominal fullness, early satiety, a pelvic mass and ascites is typical for women with EOC, unfortunately however, these symptoms often present only when the disease is advanced in stage. A screening test to detect early stage ovarian cancer, when the 5-year survival is nearly 70–90% [13], has yet to be developed. The low incidence of early stage EOC precludes extensive study of disease pathogenesis, thus most research occurs in late stage disease. We identified women with advanced EOC and collected paired ascites and TIF from ovarian cancer tumors from each patient for proteomic analysis. Ascites and TIF were immunodepleted of highly abundant proteins prior to LC-MS/MS analysis. Our data revealed that ascites and TIF harbor very different protein profiles. Ascites possesses proteomic characteristics similar to that of serum, being composed largely of proteins derived from the extracellular compartment. Conversely, TIF is comprised of proteins more representative of a tissue microenvironment, with many derived from various compartments from cells.

In our analysis, 569 proteins were identified in common between all patient TIF samples, compared to 171 proteins commonly identified in corresponding ascites fluid. Among these, PRDX1 was identified in greater abundance in TIF samples as compared to ascites. TIF is hypothesized to contain a higher relative concentration of proteins derived through passive/enzymatic shedding, secretion, efflux and apoptosis. In a similar fashion, these same processes likely result in “leakage” of some or all of these same proteins into the vasculature and peripheral circulation. The observed greater level of PRDX1 in TIF relative to ascites is not unexpected for manifold biologically- and analytically-related reasons. From a biological standpoint, TIF is largely derived from the tumor microenvironment and hence likely to contain higher relative concentrations of tumor/stroma-derived proteins as compared to other peripheral body fluids, such as ascites and serum. The consequence of this difference in dynamic range of protein concentration between these body fluids effectively lowers the dynamic range of protein concentration in ascites and/or serum as compared to TIF. For these reasons, inclusion of proximal fluids such as TIF in proteomic-based biomarker discovery investigations represents a novel strategy for harvesting shed and secreted proteins proximal to the tumor tissue microenvironment whose presence is likely to be reflected in serum from the same patients.

PRDX1 is a ubiquitous tissue-derived protein with known function as an antioxidant. PRDX1 primarily protects cells from DNA damage and potential mutagenesis by forming alcohols following enzymatic reaction with reactive oxygen species (ROS), such as hydrogen peroxide and hydroxyl radicals [14]. PRDX1 is of particular interest due to its known elevated level of gene expression in numerous cancers [15], [16]. PRDX1 presence in carcinomas is associated with inhibition of apoptosis, equating to increased tumor survival [16], [17], [18]. While there is limited data with respect to PRDX1 and its effects on EOC pathogenesis and clinical outcomes, Chung et al. have proposed that PRDX1 expression in EOC is a prognostic indicator of decreased survival [19]. In addition, several other peroxiredoxin family members (PRDX2-6) are thought to be linked to ovarian carcinogenesis, where these have been reported to be elevated in borderline ovarian tumors compared to benign ovarian lesions [20]. Maxwell et al. recently conducted a large scale proteomic analysis of laser microdissected endometrial cancer tissue and noted that even stage I endometrial cancers had elevated levels of proteins involved in oxidative stress and inflammation compared to normal endometrium. PRDX1 was among the oxidative stress proteins that was validated to be significantly elevated in stage I endometrial cancer tissue [21]. In the present study, the presence of PRDX1 in TIF and ascites from ovarian cancer patients was verified by western blot to be elevated in abundance in TIF as compared to ascites. Based on the hypothesis that a subset of TIF proteins represent shed/secreted proteins from the microenvironment and hence may be exchangeable, and hence detectable, within peripheral blood, we attempted to determine the levels of PRDX1 in blood serum using a highly validated, commercially available ELISA. These results demonstrated that patients with advanced EOC had a 6-fold elevated level of PRDX1 in their serum compared to patients with normal/benign ovaries (p = 0.0188).

Although PRDX1 has not been reported previously to be present in serum, based on the hypothesis that some (if not all) TIF proteins are exchangeable with peripheral circulation, we utilized a targeted ELISA-based approach to first verify this hypothesis and secondarily to ask the question whether this protein is differentially abundant in serum from patients with EOC as compared to individuals with benign pathologies of the ovary. We limited this external validation of PRDX1 to serum because elevated levels of PRDX1 have already been demonstrated in ovarian cancer tissue by western blot and immunohistochemistry [19]. Ideally, panels of selected biomarkers are likely needed in order to develop a validated screening test for early EOC detection [22], our sole focus was to demonstrate the feasibility of translating MS-based proteomic discoveries from TIF into targeted serum-based assays, in this case PRDX1. We acknowledge that we cannot conclude that PRDX1 is sufficiently sensitive and specific to be considered as a screening biomarker for EOC based on our limited validation sample cohort, however, that it is detectable and elevated in ovarian cancer patient serum compared matched benign controls provides further supporting evidence to the hypothesis that protein candidates discovered in TIF are likely to be present in peripheral circulation.

## Materials and Methods

### Patient Selection

Research was conducted under the University of Pittsburgh Institutional Review Board approved protocol #IRB0406147. All samples were obtained after review of written informed consent and were de-identified upon collection and analyzed anonymously. Intra-operative collection of tumor, ascites and serum were from patients with suspected ovarian cancer at the time of their initial surgery and occurred within 30 min of surgical removal. All patients were chemotherapy and radiation therapy naïve. Patients with advanced EOC (stage IIC or greater) and pathology-proven papillary serous ovarian carcinoma subtype were selected for sample processing and analysis. Serum used for validation of PRDX1 was derived from an internal database of de-identified patients classified as having EOC or benign ovarian pathology. Clinicopathological details of these patients were limited to their gynecologic history.

### Sample Processing

For the processing of TIF, approximately 0.25–0.50 gram wet-weight of tissue obtained at surgery was immediately washed twice for 5 min with 5 mL of phosphate buffered saline (PBS). The tissues were then incubated in 1 mL of PBS for 1 h at 37°C. Following incubation, tissue was removed and the TIF supernatant was clarified of cellular debris by centrifugation at 1000*× g* for 2 min and stored at −80°C. Ascites samples were aliquotted into microcentrifuge tubes, clarified at 1000*× g* for 2 min and stored at −80°C. Total protein in the TIF and ascites samples was quantified by the bicinchoninic (BCA) assay (Pierce, Rockville, IL). Samples were concentrated using Microcon-3 centrifugal filter devices (Millipore, Billerica, MA) according to the manufacturer's instructions and subjected to immunodepletion of the 14 most abundant serum proteins using the Multiple Affinity Removal System (Hu-14, Agilent Technologies, Inc., Santa Clara, CA) according to the manufacturer's protocol, with the substitution of a 4× sample dilution buffer rather than the 1× buffer to prevent potential sample loss due to extensive concentration of the TIF and ascites. The depleted TIF and ascites samples were exchanged into 25 mM ammonium bicarbonate and protein was quantified by BCA assay. Corresponding blood samples were collected in red-top vacutainer venous collection tubes (Becton, Dickson and Company, Franklin Lakes, NJ), permitted to clot at room temperature for 45 min, centrifuged at 2200*× g* for 10 min to collect serum and aliquotted and stored at −80°C.

### In-gel Digestion Protocol

Ten micrograms each of ascites or TIF were resolved by 1D-PAGE and gel bands were visualized by Coomassie blue. Five gel bands were excised evenly across all samples and destained in 25 mM ammonium bicarbonate, 50% ACN. Gel slices were reduced with 10 mM DTT at 56°C for 1 h followed by alkylation with 55 mM iodoacetamide for 45 min at ambient temperature in the dark. Samples were digested with trypsin overnight and peptides were extracted from gel bands with 70% ACN/5% formic acid. All samples were lyophilized to dryness and resuspended in 0.1% trifluoroacetic acid prior to analysis by liquid chromatography (LC)-tandem mass spectrometry (MS/MS).

### Mass Spectrometry and Bioinformatic Analyses

The peptide extracts from each sample gel band were analyzed in duplicate on a Dionex Ultimate 3000 liquid chromatography system (Dionex, Sunnyvale, CA) coupled online via electrospray ionization to a linear ion trap (LTQ) MS (ThermoFisher Scientific, San Jose, CA). Separations were performed using 75 µm i.d.×360 µm o.d.×20 cm long fused silica capillary columns (Polymicro Technologies, Phoenix, AZ) slurry packed in house with 5 µm, 300 Å pore size C-18 silica-bonded stationary phase (Jupiter, Phenomenex, Torrance, CA). Following sample injection onto a C-18 precolumn (Dionex), the column was washed for 3 min with mobile phase A (2% acetonitrile, 0.1% formic acid) at a flow rate of 30 µL/min. Peptides were eluted using a linear gradient of 0.33% mobile phase B (0.1% formic acid in acetonitrile)/minute for 130 min, then to 95% B in an additional 15 min, all at a constant flow rate of 200 nL/min. Column washing was performed at 95% B for 15 min for all analyses after which the column was re-equilibrated in mobile phase A prior to subsequent injections. The MS was operated in a data-dependent MS/MS mode in which each full MS scan was followed by seven MS/MS scans performed in the linear ion trap (LIT) where the seven most abundant peptide molecular ions were selected for collision-induced dissociation (CID) using a normalized collision energy of 35%. Data were collected over a broad precursor ion selection scan range (*m/z* 375–1800) and dynamic exclusion was enabled to minimize redundant selection of peptides previously selected for CID.

Tandem mass spectra were searched against the UniProt human protein database (11/09 release) from the European Bioinformatics Institute (http://www.ebi.ac.uk/integr8), using SEQUEST (Bioworks 3.3.1, ThermoFisher Scientific). Search criteria were set as follows: peptides were searched fully tryptic with up to two missed cleavage sites, dynamic modifications of methionine oxidation (15.9949 Da) and cysteine carboxyamidomethylation (57.0215 Da), precursor mass tolerance of 1.4 Da and fragment ion tolerance of 0.5 Da. Peptides were considered legitimately identified if they met specific charge state and proteolytic cleavage-dependent cross correlation scores of 1.9 for [M+H]^1+^, 2.2 for [M+2H]^2+^ and 3.5 for [M+3H]^3+^, and a minimum delta correlation of 0.08. A false peptide discovery rate of less than 2% was determined by searching the primary tandem MS data using the same criteria against a decoy database wherein the protein sequences are reversed [5]. Results were further filtered using software developed in-house, and differences in protein abundance between samples were derived by summing the total CID events that resulted in a positively identified peptide for a given protein accession across all samples (spectral counting) [23].

### Western Blot Analysis

Ten µg of total protein from each sample was resolved by 1D-PAGE using 4–12% Bis-Tris or 7% Tris-acetate NuPAGE gels (Invitrogen Carlsbad, CA) and transferred to Immobilon-PSQ PVDF membranes (Millipore) using the Invitrogen Xcell II Blot Module according the manufacturer's protocol. Primary antibody was rat monoclonal anti-human PRDX1 (Abcam, Cambridge, MA) and secondary antibody was horseradish peroxidase-conjugated donkey anti-rat (Abcam Cambridge, MA), pre-absorbed with serum. Membranes were blocked with 0.5% dried milk with TBS with 0.1% Tween-20 (TBST) as the diluent. Primary antibody were applied at 1∶1000 in TBST and membranes were incubated overnight at 4°C. Membranes were washed with TBST and incubated with secondary antibody (1∶75,000 dilution) for 1 h at ambient temperature. Membranes were washed with TBST and incubated with Pico Super Signal ECL (ThermoFisher) for 5 min prior to chemiluminescent exposure.

### Peroxiredoxin 1 ELISA

The levels of abundance of PRDX1 in patient sera were measured using the peroxiredoxin 1 Human ELISA kit (BioVendor, Candler, NC) according to the manufacturer's instructions. Serum samples from patients with ovarian cancer and benign pathology were diluted 1∶3 prior to measurement and were assayed simultaneously and in duplicate. Serial dilutions of PRDX1 standard were assayed in parallel with serum samples. The optical density was plotted against standard PRDX1 concentrations to generate the standard curve according to the manufacturer's protocol. A non-parametric Wilcoxon rank-sum test was used to determine the significance of the difference between the median PDRX1 quantities in sera of patients with benign ovarian pathology versus patients with advanced EOC. The null hypothesis was that data in the two observation groups are independent samples from identical continuous distributions with equal medians against the alternative hypothesis, that the groups do not have equal medians. Significance was accepted at a two-tailed p value<0.05.

## Supporting Information

Table S1 **Proteins identified from four epithelial ovarian cancer patient matched ascites and tissue interstitial fluid (TIF).** The list of protein identifications (rows) represent the summed spectral counts (values) from analyses of ascites and TIF, each of which were resolved by denaturing gel electrophoresis from which five gel bands were in gel digested and analyzed in duplicate by liquid chromatography-tandem mass spectrometry.
(XLS)
